# Propofol + Granisetron vs. Propofol + Metoclopramide in Symptom Management of Acute Migraine Headache; a Double-Blind Randomized Clinical Trial 

**DOI:** 10.22037/aaem.v10i1.1561

**Published:** 2022-03-05

**Authors:** Samaneh Abiri, Mehdi Chegin, Reza Soleimani, Naser Hatami, Navid Kalani, Esmail Rayatdoost

**Affiliations:** 1 Department of Emergency Medicine, Research Center for Noncommunicable Diseases, Jahrom University of Medical Sciences, Jahrom, Iran.; 2 Student Research Committee, Jahrom University of Medical Sciences, Jahrom, Iran.; 3 Research Center for Social Determinants of Health, Jahrom University of Medical Sciences, Jahrom, Iran.

**Keywords:** Propofol, granisetron, metoclopramide, migraine disorders

## Abstract

**Introduction:**

Acute headache is one of the most common reasons for emergency department (ED) visits. This study aimed to compare the combination of propofol and granisetron with propofol and metoclopramide in symptom management of acute migraine headache.

**Methods:**

In this double-blind randomized clinical trial, 60 adult patients with acute migraine headache who referred to ED were randomly divided into two groups of propofol + metoclopramide and propofol + granisetron. Pain and nausea/vomiting severity as well as blood pressure were compared between groups 30, 45, and 60 minutes after treatment.

**Results:**

The two groups had similar situation regarding mean age (p = 0.606), sex distribution (p = 0.793), baseline severity of pain (p = 0.642), frequency of nausea/vomiting (p = 0.488), and vital signs (p > 0.05). The severity of pain was similar in the two groups 30 (p = 0.731), 45 (p = 0.460), and 60 (p = 0.712) minutes after treatment. The number of patients with resistant nausea and vomiting 60 minutes after treatment was significantly higher in metoclopramide group (30.0% versus 10.0%; p = 0.033). Diastolic pressure 60 minutes after treatment (81.43 ±8.94 vs. 74.97 ± 4.8; p = 0.001) and heart rate 30 minutes after treatment (68.87 ±6.52 vs. 73.57± 7.62; p = 0.013) had statistically significant differences between the groups.

**Conclusion:**

The combination of propofol and granisetron was superior to propofol and metoclopramide in case of controlling nausea and vomiting of cases with acute migraine headache; meanwhile, no differences were observed in case of pain relief and hemodynamic status between the two groups.

## 1. Introduction:

Headache is one of the most common types of pain that make many patients go to pain clinics or emergency departments (ED). In the United States, 90% of the population experienced a headache, 50% of the population suffered from one type of headache, and 25% experienced recurrent and debilitating attacks (1). According to a study done in the United States, headaches cost the US economy more than $50 billion a year, resulting in decreased work and educational efficiency as well as a significant increase in medical expenditures for the US health system. 

Migraine is the most frequent form of headache, and it can cause incapacity, as well as a loss of financial resources and a reduction in the efficiency of work and education. At least once a year, 15-20 percent of women and 6-10 percent of men suffer from migraines. It has been revealed that the age ranges of 25-55 years have the highest occurrence (2). Migraine disorders, including classic migraine, are caused by spasms of cerebral arteries, and migraine pain is caused by subsequent dilation of extra cranial arteries (3). Migraine headaches often present as one-sided, throbbing pains and are accompanied by nausea and vomiting, sensitivity to light, and fatigue (4-6). Current prophylactic treatments usually include dopamine receptor antagonists such as prochlorperazine or metoclopramide, which are often combined with diphenhydramine. Studies have shown that these drugs are safer and more effective than nonsteroidal anti-inflammatory drugs and sumatriptan (7-10). Metoclopramide stops the effects of dopamine on the central nervous system and other organs. Its effects on the medulla oblongata (CTZ) region suggest that it is a beneficial antiemetic for nausea and vomiting, while it may be an 5-HT3 receptor antagonist. It should also be noted that granisetron is a potent selective 5-HT3 antagonist that is primarily used to treat nausea due to chemotherapy. Side effects include headache, diarrhea, constipation, anxiety, and insomnia (12). Another recommended treatment for acute migraine is an anesthetic agent called propofol. The mechanism of action of propofol is through its agonist activity on the gamma-Aminobutyric acid (GABA) aminobutyric acid (GABAA) beta 1 subunit, which leads to hyperpolarization and inhibition of neuronal stimulation (13). This study aimed to compare the use of propofol + granisetron with propofol + metoclopramide in symptom management of patients who referred to the emergency department (ED) with migraine headache.

## 2. Methods:

***2.1. Study design and setting***

The present study is a double-blind randomized clinical trial that was performed during a one-year period from June 2018 to June 2019 on patients with acute migraine headache who referred to ED of Peymaniyeh Hospital, Jahrom, Iran. Before the patients were included in this study, the research process was explained and informed consent was obtained from them. Throughout the study, researchers adhered to the principles of Helsinki Declaration and confidentiality of patient information. All costs of the project were covered by the researchers and no additional costs were incurred by the patients. This study has been approved by the ethics committee of Jahrom University of Medical Sciences under the ethical code IR.JUMS.REC.1397.060 and has been registered in the Iranian registry of clinical trials under the number IRCT20201003048903N2. (Http: //www.irct .ir).

***2.2. Participants***

Adult patients (18 years old or older) without history of head trauma in recent months, no abnormal neurological findings, and no hearing or verbal impairment with the criteria of migraine headache based on International Headache Society's definition (2 to 8 headache episodes per month in the previous 3 months, with or without aura) were included. Patients with moderate to severe pain (Visual Analogue Scale (VAS) > 3) were included. Patients who had taken other medication for pain relief before presenting to the ED, those with history of allergy to the studied drugs, pregnant cases, cases with unstable hemodynamic or loss of consciousness, and finally cases not agreeing to enter the study were excluded (14).

***2.3. Intervention***

Patients were randomly divided into 2 groups: 1) propofol and granisetron and 2) propofol and metoclopramide, using simple random sampling method by tossing coins. The person performing the work steps, the person collecting the information, and the patient were unaware of the type of drug used. 

All patients were under close clinical, electrocardiographic, pulse oximetry, and non-invasive blood pressure monitoring during the procedure. Patients in group one received intravenous (IV) bolus dose of 2 mg granisetron (Caspian Tamin Pharmaceutical Co., Iran) over 3 minutes plus 10 mg propofol 1% (Aram Pharmaceutical Co.,Ltd , Iran) over 3 minutes (every 5 to 10 minutes up to a maximum dose of 80 mg) through cephalic, basilic, or any available superficial veins of the hands; patients in group two received 10 mg metoclopramide (Caspian Tamin Pharmaceutical Co., Iran) over 3 minutes plus 10 mg propofol 1% over 3 minutes (every 5 to 10 minutes up to a maximum dose of 80 mg). Propofol injections continued until the headache resolved. 

***2.4. Outcomes***

Pain and nausea/vomiting management were considered as the primary measured outcomes of the study. Hemodynamic changes (blood pressure and heart rate) were considered as the secondary outcomes.

Pain and nausea/vomiting severity were measured before the injection of the drugs and at intervals of 30, 45 and 60 minutes after the injection. Nurses were blinded to type of treatment as medications were provided in syringes in packets and also patients did not know the exact medication type. We planned pethidine administration as the rescue treatment in cases of resistant pain. 

***2.5. Data collection***

The checklist designed by the researcher included demographic information (age, sex), history of headache, severity of pain, nausea and vomiting score, and vital signs (systolic and diastolic blood pressure, heart rate). 

The severity of pain was assessed using a visual acuity pain score, in which zero indicates no pain and ten indicates unimaginable pain. 

Also, the score of nausea and vomiting (0 = without nausea and vomiting, 1 = mild nausea without the need for treatment or curable nausea, 2 = nausea that can be relieved with anti-nausea medication or treatable nausea, 3= interactable nausea and vomiting) was used for measuring the severity at baseline and 60 minutes after the treatment. 

***2.6. Data analysis***

Data analysis was performed using SPSS software version 21 and intention to treat analysis. Data were reported using mean ± standard deviation or frequency (percentage). Repeated measurement Anova, chi-square, and independent t were used for comparisons. P <0.05 was considered as level of significance. Also, we calculated number needed to treat (NNT) and absolute risk reduction (ARR)(15). 

## 3. Results:

***3.1. Baseline characteristics***

60 cases with acute migraine headache were randomly divided into two 30-case groups. Table 1 compares the baseline characteristics between groups. The two groups were similar regarding mean age (47.43±15.25 in granisetron and 48.80 ±13.38 years in metoclopramide group; p = 0.606), sex distribution (p = 0.793), history of headache (p = 0.606), baseline severity of pain (p = 0.642), and vital signs (p > 0.05). Regarding nausea and vomiting, 4 patients in granisetron group and 6 patients in metoclopramide had no nausea and vomiting; while other patients needed treatment for nausea and vomiting based on our scoring (p=0.488). 

**3.2. Outcomes:**

Table 2 compares the outcomes between the two studied groups at different times after injections. 

The severity of pain was similar in the two groups at 30 (p = 0.731), 45 (p = 0.460), and 60 (p = 0.712) minutes after treatment. The number of patients with resistant nausea and vomiting 60 minutes after treatment was significantly higher in metoclopramide group (30.0% versus 10.0%; p = 0.033). Although there was a statistically significant difference between the groups regarding diastolic pressure 60 minutes after treatment (81.43 ±8.94 vs. 74.97 ± 4.8; p = 0.001) and heart rate 30 minutes after treatment (68.87 ±6.52 vs. 73.57± 7.62; p = 0.013), these differences were not clinically important. 

The pain was relieved in all cases and no case needed rescue treatment in the two groups. In granisetron group, intractable or resistant vomiting rate in 60 minutes was less than metoclopramide group (10% vs. 30%) with NNT of 5 (ARR=0.2). 

## 4. Discussion:

The results of this study showed that the combination of propofol and granisetron has a similar effect to propofol and metoclopramide regarding pain management and a superior effect regarding controlling nausea/vomiting after 60 minutes in patients presenting to ED following acute migraine headache. The two combinations had similar effects on vital signs.

The effects of metoclopramide and granisetron on migraine headaches and nausea in migraine sufferers were examined by Amiri et al. The level of pain was recorded using the VRS scale at intervals of 1 to 4 hours in this study, and the patients' nausea and vomiting condition was also assessed. The findings of Amiri et al.’s study were similar to ours in that the incidence of nausea and vomiting in the granisetron group was considerably lower than in the metoclopramide group at all time intervals after taking the medicine. Similar to our study, there was no significant difference in the incidence of headache between the two study groups (16). ‎

Medications used to treat migraines fall into two groups: suppressive or preventive. There are many options for treating acute migraine. In the present study, we focused on the combination of metoclopramide and granisetron with propofol for the treatment of headache, nausea, and vomiting in an acute migraine attack. Metoclopramide is an antiemetic agent that blocks dopamine and serotonin receptors in the CNS chemoreceptor hub receptor. Granisetron is a selective 5-HT3 antagonist that binds to receptors in the peripheral and central nervous systems with primary effects on the Golgi apparatus (17).

Leyasin et al. concluded that granisetron and metoclopramide gel have similar effects in the management of postoperative nausea and vomiting in obstetric and gynecological surgeries and that granisetron has no superiority over metoclopramide in prevention of nausea and vomiting. In contrast, we found granisetron to be better than metoclopramide in treating migraine pain (18).

Bojan Bagi and colleagues compared the effect of dexamethasone in combination with metoclopramide and granisetron on postoperative nausea and vomiting. The results of their study showed that the incidence of nausea and vomiting in the group receiving the combination of dexamethasone with metoclopramide was not significantly different from the group receiving granisetron. In fact, in this study, the effects of dexamethasone in combination with metoclopramide and granisetron in controlling nausea and vomiting after surgery were very similar and this study showed that the effectiveness of dexamethasone in combination with metoclopramide is not low compared with granisetron. This study was in line with the positive effects of granisetron and metoclopramide in controlling and reducing nausea and vomiting (19). Akerman et al. studied the effect of metoclopramide. It inhibits the excitability of vascular neurons, which is highly predictive of the anti-migraine action of this drug (20). Another study evaluated the antiemetic effects of ondansetron and granisetron in preventing postoperative nausea and vomiting in a patient undergoing laparoscopic surgery. This study showed that the incidence of severe nausea and vomiting was 7% among patients receiving intravenous granisetron, followed by 20% in the ondansetron group and 50% in the placebo group (22). The results of this study are in line with our study regarding the effect of granisetron. In our study, the number of patients with severe nausea and vomiting resistant to treatment in the granisetron group was significantly lower than the number these patients in the metoclopramide group. We have shown that the effect of granisetron on nausea and headache is relatively higher than that of metoclopramide. In line with the results of our study, in a randomized double-blind study on 100 female patients, the effect of granisetron and ondansetron in preventing nausea and vomiting was evaluated in patients undergoing elective laparoscopic cholecystectomy. There was a significant difference between the two groups and the incidence of nausea and vomiting in the granisetron receiving group was significantly lower than the incidence of nausea and vomiting in the group receiving ondansetron (23). Gauchan et al. Evaluated the antiemetic effect of ondansetron and granisetron in patients undergoing laparoscopic cholecystectomy during the first 24 hours after anesthesia. They showed that granisetron was able to effectively reduce the incidence of nausea and vomiting compared to ondansetron in the first 24 hours (24). In line with the results of the present study, in another study by Savant et al., the effects of ondansetron and granisetron on preventing nausea and vomiting in the patients undergoing oral and maxillofacial surgery were investigated. The results of this study showed that the incidence of nausea and vomiting in the granisetron group was significantly lower than ondansetron. Patients receiving granisetron showed a higher rate of a headache compared to the ondansetron group (25). In contrast, in our study the incidence of headache after drug injection was not significantly different from that of metoclopramide. In one study, Mohammed and colleagues examined the effects of metoclopramide and ondansetron on the control of postoperative nausea and vomiting. The results of their study showed that metoclopramide was more effective than ondansetron in controlling nausea and vomiting after surgery (26). This was also confirmed in Malekshoar et al.’s study(27).

**4.1. Strength and Limitations**

The strength of this study was using the combination of these medications so that a statistically and clinically significant decrease in pain score was recorded in both groups. We did not record the number of propofol doses for each group to be able to compare the groups in this regard, this might have biased the conclusion. Another limitation of this study was the low number of patients, which might be a possible reason for the absence of difference in pain reduction properties of medications. Also, we did not assess the other patient characteristics as well as any painkiller being consumed before referral and time from initiation of symptom to presentation to ED.

**Figure 1:**
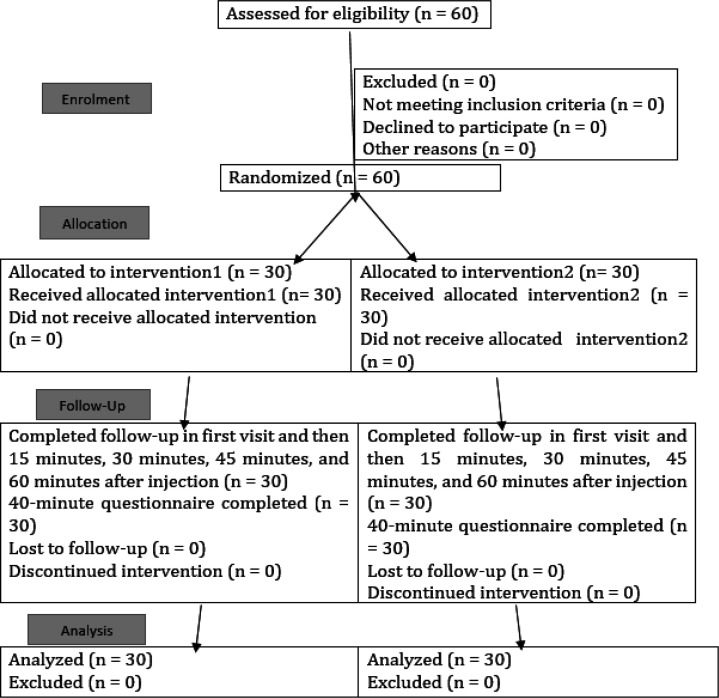
The CONSORT flow chart of the randomized trial

**Table 1:** Comparing the baseline characteristics between the two groups

Variable	**Granisetron**	**Metoclopramide**	P*
**Age (year)**			
Less than 30	3 (10.0)	1 (3.3)	0.606
30-50	14 (46.6)	16 (53.3)	
More than 50	13 (43.3)	13 (43.3)	
**Sex **			
Male	17 (56.6)	18 (60.0)	0.793
Female	13 (43.3)	12 (40.0)	
**Blood pressure (mmHg)**			
Systolic	142.7±17	139.2 ±15.5	0.108
Diastolic	73.97 ± 4.8	75.43± 8.9	0.432
**Heart rate (beats/minutes)**			
Mean ± SD	69.87±6.52	70.87±6.52	0.371
**Pain severity (VAS)**			
Mean ± SD	5.5 ±2.29	5.2 ±2.4	0.642
**Nausea/vomiting severity before treatment**			
No nausea/vomiting	4 (13.3)	6 (20.0)	0.488
Treatable	26 (86.7)	24 (80.0)	

Data are presented as mean ± standard deviation (SD) or frequency (%). VAS: visual analog scale.

**Table 2:** Comparing the outcomes between the two studied groups 30, 45, and 60 minutes after injections

**Variables**	**Granisetron**	**Metoclopramide**	**P**
**Severity of pain (VAS)**			
30 minutes	3.87 ±2.06	4.1 ±2.2	0.731
45 minutes	3.3 ± 1.56	3.6 ±1.6	0.460
60 minutes	3.6 ±1.61	3.9 ±1.8	0.712
**Systolic blood pressure (mmHg)**			
30 minutes	146.4 ±18.3	147.1 ±18.6	0.222
45 minutes	140. 4 ±15.2	140.1 ±14.9	0.344
60 minutes	145.8 ±18.5	147.1 ±18.6	0.113
**Diastolic blood pressure (mmHg)**			
30 minutes	75.96 ± 4.79	77.43 ±8.94	0.433
45 minutes	76.97 ±4.8	78.43 ±8.94	0.188
60 minutes	74.97 ± 4.8	81.43 ±8.94	0.001
**Heart rate (beat/minute)**			
30 minutes	68.87 ±6.52	73.57± 7.62	0.013
45 minutes	69.87±6.52	71.87±6.52	0.146
60 minutes	73.57±7.62	72.57±7.62	0.704
**Nausea/vomiting 60 minutes after injection**			
Mild	13 (43.3)	3 (10.0)	0.033
Curable	7 (23.3)	9 (30.0)	
Treatable	3 (10.0)	3 (10.0)	
Resistant	3 (10.0)	9 (30.0)	

Data are presented as mean ± standard deviation or frequency (%). VAS: visual analog scale**. **

## 5. Conclusion:

The results of this study showed that the effect of propofol and granisetron combination was similar to propofol and metoclopramide combination regarding pain management; but it had a superior effect regarding controlling nausea/vomiting after 60 minutes in patients presenting to ED following acute migraine headache. Both combinations had similar effects on vital signs.

## 6. Declarations:

### 6.1. Acknowledgement

We would like to thank the Clinical Research Development Unit of Peymanieh Educational and Research and Therapeutic Center of Jahrom University of Medical Sciences for providing facilities to this work.

### 6.2. Authors Contributions

ER conceptualized the study design and RS and NK wrote the study protocol. RS and NK collected the data and analyzed it. Manuscript was drafted by NK and ER. All authors contributed to the revisions. 

### 6.3. Conflict of interest

There are no conflicts of interest in this study.

### 6.4. Funding

The research was financially supported by Jahrom University of Medical Sciences.
